# Comparison of global and regional myocardial blood flow quantification using dynamic solid-state detector SPECT and Tc-99 m-sestamibi or Tc-99 m-tetrofosmin in a routine clinical setting

**DOI:** 10.1007/s10554-025-03339-4

**Published:** 2025-01-30

**Authors:** Wiebke Wieting, Frank M. Bengel, Johanna Diekmann

**Affiliations:** Department of Nuclear Medicine, Hannover Medical School, Carl-Neuberg-Str. 1, 30625 Hannover, Germany

**Keywords:** Absolute quantitative SPECT, Myocardial perfusion imaging, Myocardial blood flow, Myocardial flow reserve

## Abstract

Solid-state detector single photon emission computed tomography (SPECT) enables the acquisition of dynamic data for calculation of myocardial blood flow (MBF) and myocardial flow reserve (MFR). Here, we report about our experiences on routine clinical use and robustness using Tc-99 m-sestamibi and Tc-99 m-tetrofosmin. 307 patients underwent dynamic list-mode myocardial perfusion imaging (MPI) and standard static MPI for clinical workup of coronary artery disease on a dedicated cardiac SPECT camera. After exclusion of 33 scans, 274 scans were eligible for MBF and MFR calculation using a 1‐tissue‐compartment model. Attenuation correction was performed for all patients using an external computed tomogram. Patients underwent stress-only scans, both stress and rest scans or rest-only scans using Tc-99 m-tetrofosmin or Tc-99 m-sestamibi. 30 patients without known cardiovascular comorbidities and without perfusion defect on static scans were compared in a sub analysis. Global stress myocardial blood flow (MBF) was significantly higher than rest MBF (2.3 vs. 1.1 ml/min/g; p < 0.001), and showed a high variability among individuals. Global myocardial flow reserve (MFR) was 2.1 (range 0.5–7.8). An analysis of 30 patients without known cardiovascular comorbidities yielded similar stress MBF measures for Tc-99 m-sestamibi and Tc-99 m-tetrofosmin (3.1 ± 1.2 vs. 2.8 ± 0.9 ml/min/g; p = 0.429). The use of attenuation correction lead to systematically lower MBF measures. Patients who underwent a one-day protocol had notably higher rest MBF (1.2 ± 0.5 vs. 1.0 ± 0.46 ml/min/g; p = 0.009) and consequently a lower MFR. Summed defect scores from standard static scans and presence of cardiovascular comorbidities negatively impacted MBF and MFR. Quantitative SPECT MBF and MFR in a clinical routine setting yields flow measures in range of expectation at an albeit wide range and is comprehensibly linked with results from standard static scan and patients history of cardiovascular diseases. Use of one-day protocols and attenuation correction systematically alters quantitative results. However, SPECT-derived MBF and MFR lack clinical reliability due to less validated reference ranges and high inter-individual variability.

**Graphical abstract:**

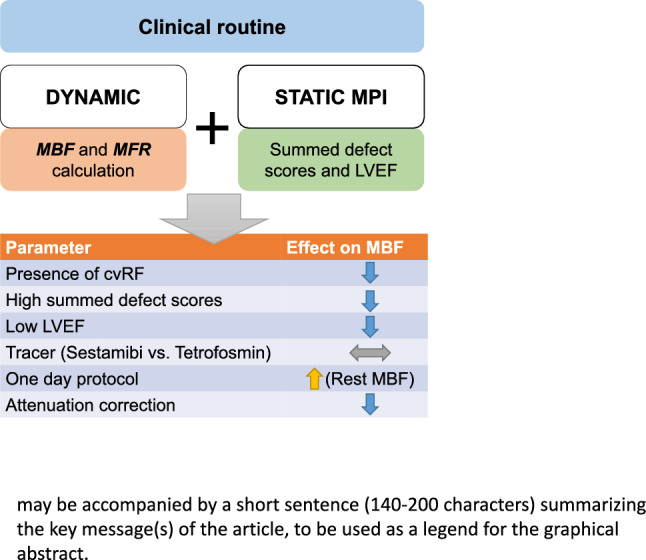

## Introduction

Quantitative measurements of myocardial blood flow (MBF) and myocardial flow reserve (MFR) derived from positron emission tomography (PET) are readily obtained and have been shown to provide diagnostic and prognostic benefit [1–8]. Therefore, quantitative perfusion assessment from PET has entered clinical routine.

Static myocardial perfusion imaging (MPI) using single photon emission computed tomography (SPECT) is the clinical gold standard and the most widely used tool for evaluation of myocardial perfusion in nuclear medicine. Quantitative perfusion analysis using SPECT is possible but has not been widely established yet due to technically demanding methodology. Semiconductor cameras with a high temporal resolution and improved count density enable dynamic list-mode acquisition. Initially, feasibility of quantitative myocardial blood flow (MBF) and myocardial flow reserve (MFR) calculation was demonstrated using conventional SPECT cameras [9–13]. In a porcine model, MBF and MFR results for three standard SPECT tracers (Tl-201, Tc-99 m-tetrofosmin and Tc-99 m-sestamibi) acquired on a dedicated SPECT camera correlated well with results from microsphere-derived flow [8]. After first proof of feasibility [14], multiple studies have validated MBF measures by direct comparison to angiographic findings [15–20] and demonstrated a prognostic value [21]. Added value of MBF calculation has been reported for microcirculation and multivessel disease [22, 23].

However, the real-world feasibility and usefulness of CZT-SPECT-derived MBF and MFR needs to be supported by more reports from standard clinical settings with diverse patient populations. The added clinical and prognostic value of PET-derived MBF and MFR has been imposingly demonstrated [24]. Here, we summarize our initial experience measuring MBF and MFR derived from solid-state detector SPECT in clinical practice using Tc-99 m-sestamibi and Tc-99 m-tetrofosmin.

## Materials and methods

### Study population

**Fig. 1:**
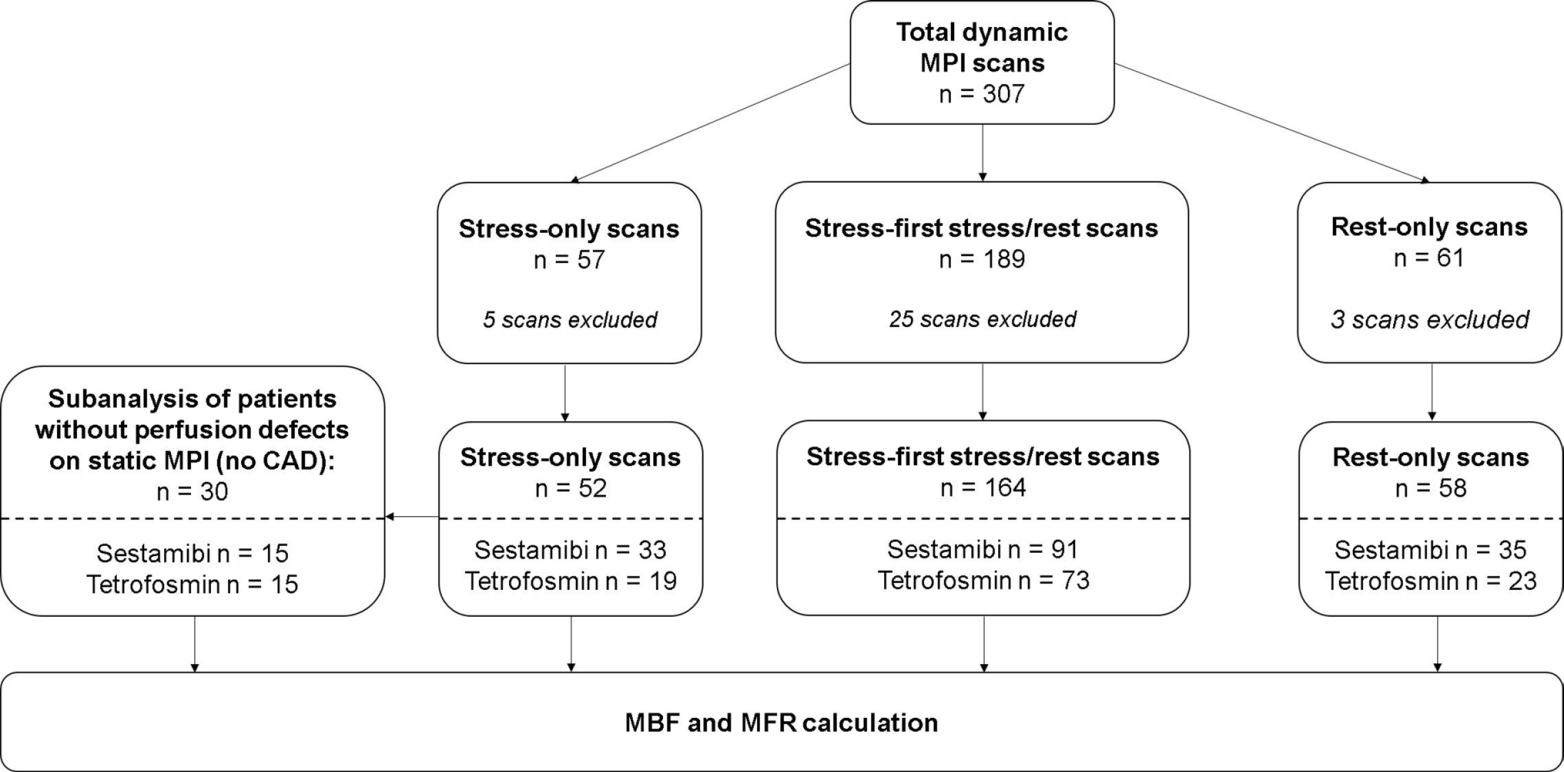
The study flow chart gives an overview of the included dynamic perfusion scans for calculation of myocardial blood flow (MBF) and myocardial flow reserve (MFR). Scans are divided in three groups: stress-only stress / rest and rest-only scans. Total numbers and specific numbers for Tc-99 m-Sestamibi and Tc-99 m-Tetrofosmin scans are given. Reasons for exclusion of scans are described in the methods

307 patients who were referred for the clinical workup of coronary artery disease (CAD) underwent dynamic and static myocardial perfusion imaging from June 2017 to December 2020. Dynamic and standard static scans were conducted in clinical routine, based on camera availability without any other preselection. All patients gave written informed consent prior to imaging. Based on clinical indication patients underwent either stress-only (n = 57), stress-first two-day (n = 99), stress-first one-day (n = 90, total stress scans n = 189) or rest-only protocols (n = 61, Fig. 1). Of the initial 307 patients, 33 had to be excluded leaving 274 scans for final analysis. Exclusions were necessary due to high spillover from infradiaphragmatic activity (n = 21), inconclusive static imaging results (negative summed difference score, n = 8) or other technical difficulties in image acquisition (n = 4). For a subanalysis, only patients without static scan defects and a negative cardiovascular history were included and 15 Tc-99 m-tetrofosmin and 15 Tc-99 m-sestamibi were compared (30 patients total).

The study design and its implementation were approved by the local ethical committee.

### Dynamic, static and gated SPECT and low-dose CT data acquisition

**Fig. 2:**
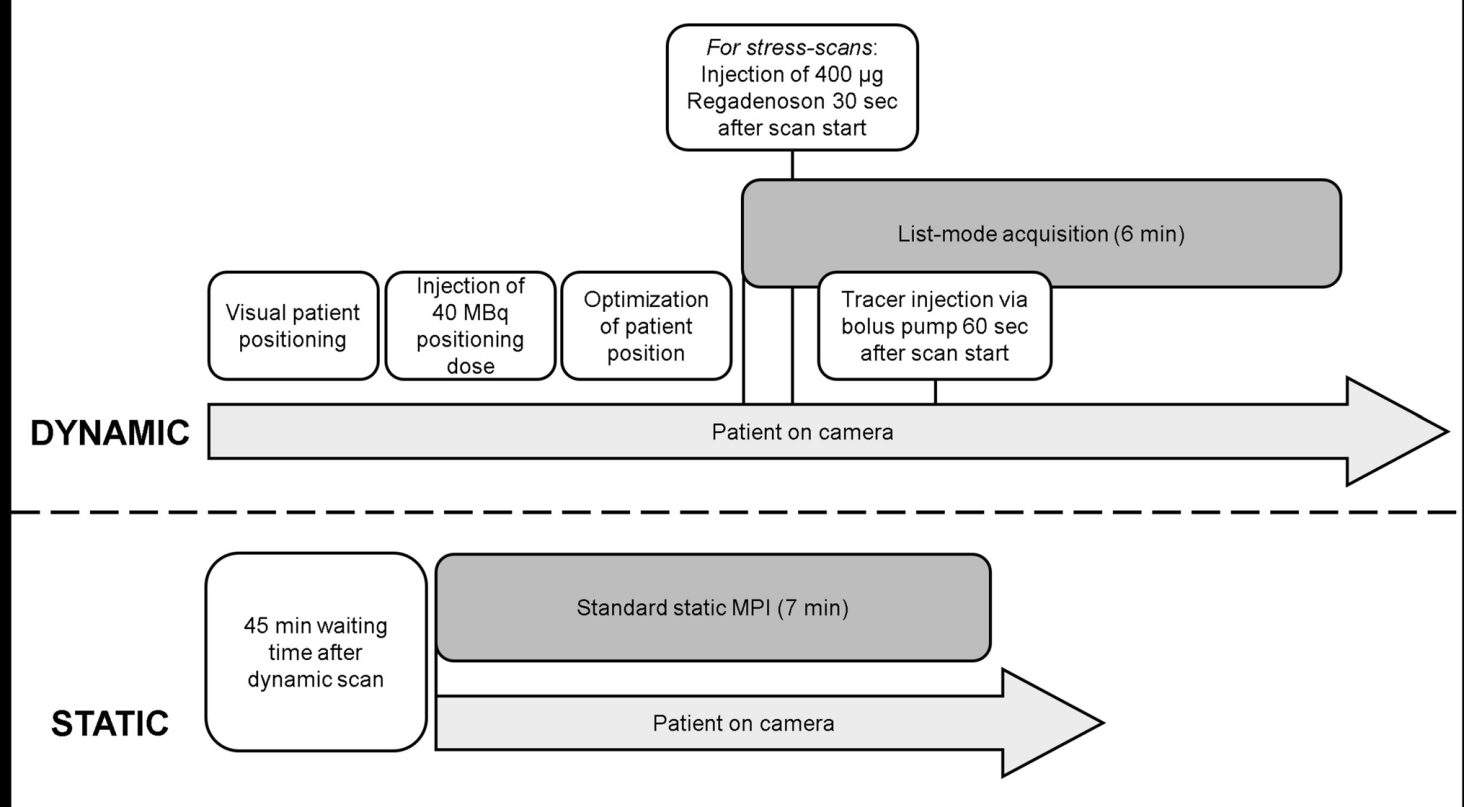
Timeline for dynamic and static image acquisition. Dynamic scan: First, patients are visually positioned under the CZT camera, then a 40 MBq tracer test dose is intravenously injected. After optimizing patient position, dynamic list mode acquisition is started (duration 6 min). Static scan: After a break of 45 min according to our clinical standards, a seven min static scan is additionally acquired

Patients were positioned in a supine position with the heart centered in the field of view and with arms above their head without prior tracer injection. For stress SPECT imaging patients abstained from caffeine for 24 h. The used SPECT workflow is depicted in Fig. 2. After injection of a test dose of 40 MBq Tc-99 m-sestamibi or Tc-99 m-tetrofosmin, patient positioning was optimized and the heart was centered in the field of view of the CZT camera (Discovery NM 530c; GE Healthcare, Haifa, Israel). Dynamic list-mode acquisition over 6 min was started. After a 60 s prerun to monitor baseline activity, 342 ± 78 MBq Tc-99 m-sestamibi or 320 ± 80 MBq Tc-99 m-tetrofosmin for stress imaging and 489 ± 150 MBq Tc-99 m-sestamibi or 471 ± 118 MBq Tc-99 m-tetrofosmin for rest studies, were continuously injected via bolus pump over 30 s (Braun Bolus Pump FM, Germany). Mean administered stress dose for one-day protocols was 280 ± 69 MBq, rest dose was 607 ± 112 MBq. For two-day protocols mean stress dose was 390 ± 46 MBq and rest dose was 394 ± 45 MBq. For stress protocols, patients were injected with 400 µg Regadenoson at 30 s into the pre-run.

45 to 60 min after finished list-mode acquisition, seven min of standard static and gated scans were additionally acquired according to our clinical standards. For gating the raw data acquisition, a detected R-R interval was divided into eight equally spaced bins in time. The respective bins of all detected R-R intervals were summed and individually reconstructed. In the reconstructed data, contour detection of the cardiac surface of the left ventricle was performed using isocontours. The endocardial surface allowed the calculation of an inner volume of the LV for each bin. The left ventricular ejection fraction was determined from the ratio of the respective maximum and minimum volumes of the bins.

An external low dose CT for attenuation correction was conducted in all patients (120 mA, 120 keV, slice thickness 2.5 mm, 16 × 1.25 mm detector rows, standard kernel, cine mode).

### Data processing

Dynamic studies were processed using Corridor 4DM v2017 SPECT MBF software (Invia, Ann Arbor, MI) on a Xeleris 4.1 workstation (GE Healthcare, Haifa, Israel). List-mode data were resampled and reconstructed into 18 frames of 10 s duration and 6 frames of 30 s each. All datasets were reconstructed with CT-based attenuation correction (AC) and without attenuation correction (NC) using a standard iterative reconstruction algorithm provided by the manufacturer. Myocardial contours were automatically determined and manually adjusted as necessary. Manual motion correction was conducted for all dynamic frames. Residual activity was detected within the acquisition’s first 60 s and subtracted from the dynamic image. A region-of-interest (ROI) for blood-pool sampling was placed on the base of the septal wall. Global and regional time activity curves were created. Quality control included a manual control for the presence of a single bolus peak between 65–150 s without any double peaks or plateaus. The uptake rate constant (K1) was calculated based on the dynamic image series using a 1-tissue-compartment model. K1 was converted to MBF using a Renkin-Crone extraction-fraction correction function [17]. Finally, global and regional stress and rest MBF and MFR were calculated.

Separately acquired static and gated SPECT images were reconstructed with and without measured AC. Polar maps were calculated (Invia, Ann Arbor, MI) and summed stress (SSS), summed rest (SRS) and summed difference scores (SDS) were calculated using the AHA 17-segments model.

Calcium score was determined using 4DM software only in patients without iatrogenic foreign material close to the heart.

### Statistical analysis

Categorical parameters are given as number and percentage. Continuous variables are given as mean ± standard deviation (SD) or as median and interquartile range (IQR) as suitable. A two-sided p-value of < 0.05 was considered as statistically significant. Correlation between quantitative variables was calculated using the students t-test and Person chi-square. Wilcoxon test was used for the comparison of two dependent variables. All statistical analyses were performed with SPSS statistical software, version 27 (IBM Corp., Armonk, New York, United States). The graphs were created with GraphPad Prism, version 9.0.2 (GraphPad Software, San Diego, United States).

## Results

### Global and regional MBF and MFR shows high variance

Baseline patient characteristics, medical history and cardiovascular risk factors are summarized in Table 1.

**Table 1:** Baseline characteristics and static SPECT results

Parameters	all patients	n	Sestamibi	n	Tetrofosmin	n	P-value
Age (years) ± SD	64.55 ± 11.90	274	66.48 ± 11.60	159	61.88 ± 11.85	115	**0.001**
Male gender, n (%)	192 (70.1)	274	106 (66.7)	159	86 (74.8)	115	0.181
Cardiovascular risk factors, n (%)							
Arterial hypertension	185 (67.5)	274	114 (71.7)	159	71 (61.7)	115	0.090
Diabetes mellitus	101 (36.9)	274	60 (37.7)	159	41 (35.7)	115	0.800
Obesity^a^	71 (26.3)	274	41 (26.5)	159	30 (26.1)	115	1.00
Positive family history	43 (15.7)	274	28 (17.6)	159	15 (13.0)	115	0.319
Prior myocardial infarction	79 (28.8)	274	47 (29.6	159	31 (27.8)	115	0.788
Coronary bypass	42 (15.3)	274	26 (16.4)	159	16 (13.9)	115	0.614
Coronay stents	109 (39.8)	274	64 (40.3)	159	45 (39.1)	115	0.901
CAD known, n (%)							
No CAD	122(44.5)	274	73 (45.9)	159	49 (42.6)	115	0.623
One vessel CAD	46 (16.8)	274	24 (15.1)	159	22 (19.1)	115	0.415
Two vessel CAD	22 (8.0)	274	14 (8.8)	159	8 (7.0)	115	0.657
Three vessel CAD	84 (30.7)	274	48 (30.2)	159	36 (31.3)	115	0.895
Stress LVEF	58.84 ± 13.50	216	59.31 ± 15.26	124	58.21 ± 13.79	92	0.565
Rest LVEF	54.18 ± 15.18	222	54.33 ± 15.26	126	53.98 ± 15.14	96	0.867
Rest Reate pressure product	9524.51 ± 2295.40	217	9659.93 ± 2355.88	125	9104.65 ± 2182.63	92	0.078
Laboratory values							
CRP (maximum, mg/l)	32.52 ± 50.83	147	28.98 ± 50.44	77	36.41 ± 51.34	70	0.378
CK (maximum, U/l)^b^	839.53 ± 1817.65	119	913.25 ± 2038.30	67	744.54 ± 1500.40	52	0.604
S-NT-proBNP max ng/l	2493.77 ± 4194.93	60	2200.26 ± 4297.40	27	2733.91 ± 4160.30	33	0.629
*Static SPECT*							
MBq stress	332.70 ± 79.77	215	342.38 ± 78.36	124	319.51 ± 80.21	91	**0.037**
MBq rest	480.81 ± 136.93	222	488.60 ± 149.97	126	470.58 ± 117.63	96	0.317
Calcium Score, n (%)	93 (33.9)	274	55 (34.6)	159	38 (33.0)	115	0.798
Agatson Score	655.53 ± 1149.15	93	707.07 ± 1284.08	55	580.92 ± 931.35	38	0.584
Calcium Percentile	60.24 ± 34.84	93	57.87 ± 34.01	55	63.66 ± 36.19	38	0.440
Stress Defekt NC, n (%)	85 (31.0)	216	47 (37.9)	124	38 (41.3)	92	0.673
SSS NC	5.29 ± 7.60	216	4.99 ± 7.79	124	5.70 ± 7.37	92	0.502
Ruhe Defekt NC, n (%)	87 (31.8)	222	49 (38.9)	126	38 (39.6)	96	1.000
SRS NC	5.70 ± 8.32	222	4.87 ± 7.20	126	6.79 ± 9.54	96	0.100
SDS NC	3.10 ± 3.94	164	3.35 ± 4.44	91	2.78 ± 3.21	73	0.358
Stress Defekt AC, n (%)	90 (32.8)	216	46 (37.1)	124	44 (47.8)	92	0.126
SSS AC	5.43 ± 7.09	216	4.68 ± 6.88	124	6.45 ± 7.28	92	0.070
Ruhe Defekt AC, n (%)	100 (36.5)	222	54 (42.9)	126	46 (47.9)	96	0.497
SRS AC	5.91 ± 7.63	222	5.52 ± 7.44	126	6.43 ± 7.88	96	0.379
SDS AC	2.67 ± 3.51	164	2.34 ± 3.17	92	3.08 ± 3.88	73	0.176

Bold values indicate statistically significant (p < 0.05)

*SD* Standard Deviation, *CAD* Coronary Artery Disease, *LVEF* Left Ventricular Ejection Fraction, *CRP* C-Reactive Protein, *CK* Creatin Kinasis, *NT-pro-BNP* N-Terminal Pro Brain Natriuretic Peptide, *MBq* Megabecquerel, *SPECT* Single Photon Emission Computed Tomography, *NC* No Attenuation Correction, *AC* Attenuation Correction, *SSS* Summed Stress Score, *SRS* Summed Rest Score, *SDS* Summed Difference Score

Global stress MBF was significantly higher than rest MBF (stress MBF 2.3 ± 1.1 ml/min/g vs. rest MBF 1.1 ± 0.5 ml/min/g; p < 0.001, all patients). A high interindividual variance was detected. Global stress MBF ranged from 0.4 to 7.4 ml/min/g and rest MBF ranged from 0.3 to 3.7 ml/min/g. Mean calculated global MFR was 2.1 ± 1.1 (range 0.5–7.8). Correcting global rest MBF for rate pressure product (RPP) did not have significant impact on calculated MBF (1.1 ± 0.5 vs. 1.2 ± 0.6 ml/min/g, p = 0.221) and MFR (2.1 ± 1.1 vs. 1.9 ± 0.9 p = 0.302) in this cohort.

Regional MBF was determined for coronary territories and results are summarized in Table 2. Highest mean MBF and highest variance was detected in the right coronary territory. Men presented with lower stress MBF and a tendency towards lower MFR in comparison to females (2.0 ± 0.8 ml/min/g vs. 2.9 ± 1.2 ml/min/g, p < 0.001 and 1.9 ± 1.0 vs. 2.4 ± 1.4, p = 0.054) while rest MBF was similar (1.1 ± 0.5 ml/min/g vs. 1.2 ± 0.5 ml/min/g, p = 0.200).

**Table 2:** Global and regional MBF

MBF (ml/min/g)	All patients	Range	Stress-only scans	Range	Stress / rest scans	Range	Rest-only scans	Range
Stress								
Global	2.28 ± 1.05	0.44—7.43	2.98 ± 1.11	0.84—5.72	2.06 ± 0.93	0.44—7.43		
LAD	2.43 ± 1.09	0.54—6.39	3.14 ± 1.18	0.69—5.61	2.20 ± 0.95	0.54—6.39		
LCX	2.40 ± 1.05	0.54—7.79	2.96 ± 1.07	1.10—5.80	2.22 ± 0.98	0.54—7.79		
RCA	2.83 ± 1.23	0.71—8.05	3.56 ± 1.24	1.60—7.22	2.60 ± 1.14	0.71—8.05		
Rest								
Global	1.09 ± 0.49	0.27—3.71			1.14 ± 0.51	0.27—3.71	0.96 ± 0.41	0.39—2.64
LAD	1.22 ± 0.58	0.36—4.49			1.28 ± 0.62	0.39—4.49	1.12 ± 0.45	0.36—2.42
LCX	1.31 ± 0.66	0.21—3.75			1.32 ± 0.66	0.27—3.75	1.26 ± 0.66	0.21—3.41
RCA	1.48 ± 0.72	0.16—5.17			1.48 ± 0.72	0.16—5.17	1.49 ± 0.74	0.27—3.73
*MFR*	2.05 ± 1.13	0.51—7.82			2.05 ± 1.13	0.51—7.82		

*MBF* Myocardial Blood Flow, *MFR* Myocardial Flow Reserve, *LAD* Left Anterior Descending Coronary Artery, *LCX* Left Circumflex Coronary Artery, *RCA* Right Coronary Artery

### MBF and MFR are comparable for Tc-99 m-sestamibi and Tc-99 m-tetrofosmin

A total of 85 patients underwent a one-day protocol and 79 patients performed a two-day protocol. 41/85 one-day protocols and 32/79 two-day protocols were performed using Tc-99 m-tetrofosmin. 58 patients underwent rest-only studies of which 23 were performed with Tc-99 m-tetrofosmin. 19/58 stress-only studies were performed with Tc-99 m-tetrofosmin.

Global stress MBF was significantly higher when Tc-99 m-sestamibi was used (2.4 ± 1.1 ml/min/g vs. 2.1 ± 0.9 ml/min/g; p = 0.049). This was also true in a regional MBF analysis for the LAD (2.6 ± 1.2 ml/min/g vs. 2.2 ± 1.0 ml/min/g; p = 0.031) and RCA (3.0 ± 1.3 ml/min/g vs. 2.7 ± 1.1 ml/min/g; p = 0.049) territory (Table 3). No significant differences were detected for calculated global rest MBF (1.1 ± 0.4 ml/min/g vs. 1.1 ± 0.6 ml/min/g, p = 0.259) or MFR (2.2 ± 1.3 ml/min/g vs. 1.9 ± 1.0 ml/min/g; p = 0.109).

**Table 3:** Global and regional MBF Sestamibi vs. Tetrofosmin

MBF (ml/min/g)	Sestamibi	Tetrofosmin	P-value
Stress	*n* = *124*	*n* = *92*	
Global	2.40 ± 1.13	2.12 ± 0.90	**0.049**
LAD	2.57 ± 1.16	2.24 ± 0.96	**0.031**
LCX	2.50 ± 1.14	2.26 ± 0.91	0.101
RCA	2.97 ± 1.32	2.65 ± 1.08	**0.049**
Rest	*n* = *126*	*n* = *96*	
Global	1.06 ± 0.42	1.14 ± 0.57	0.259
LAD	1.19 ± 0.46	1.30 ± 0.71	0.216
LCX	1.27 ± 0.65	1.37 ± 0.67	0.262
RCA	1.41 ± 0.66	1.57 ± 0.80	0.111
*MFR*	2.17 ± 1.25 *(n* = *91)*	1.89 ± 0.96 *(n* = *73)*	0.109

Bold values indicate statistically significant (p < 0.05)

*MBF* Myocardial Blood Flow, *MFR* Myocardial Flow Reserve, *LAD* Left Anterior Descending Coronary Artery, *LCX* Left Circumflex Coronary Artery, *RCA* Right Coronary Artery

Additionally, 30 stress-only patients (15 Tc-99 m-sestamibi and 15 Tc-99 m-tetrofosmin) without known cardiovascular comorbidities and without perfusion defects on static scans were compared in a subanalysis. Here, no relevant differences in global (3.1 ± 1.2 ml/min/g vs. 2.8 ± 0.9 ml/min/g; p = 0.429) or regional stress MBF were detected.

### One-day stress first protocol yields higher global rest MBF

**Fig. 3:**
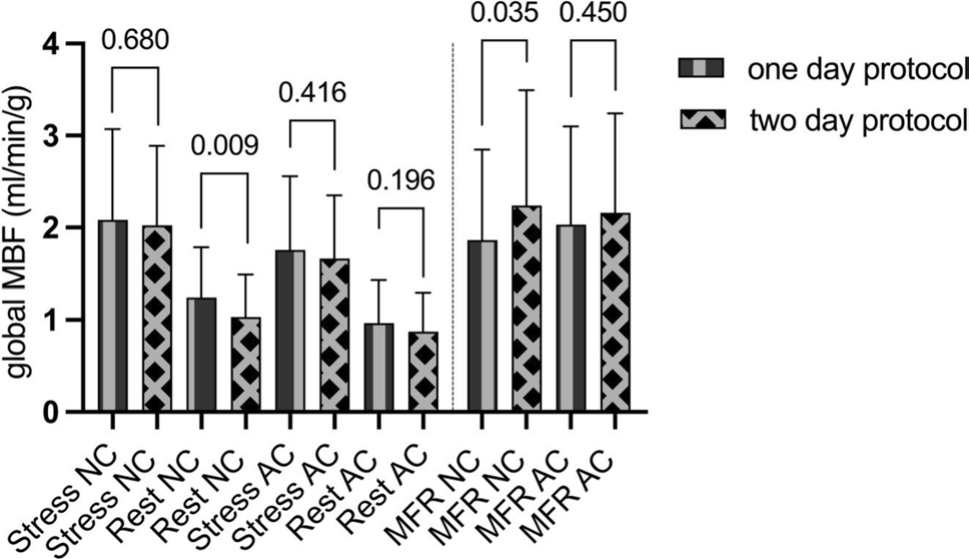
In Tc-99 m-tetrofosmin scans rest global myocardial blood flow (MBF) was significantly lower when a two day protocol was used. Consecutively, myocardial flow reserve (MFR) was higher when a two day protocol was used. Significant differences were not detectable when Tc-99 m-sestamibi was used or after attenuation correction (AC)

Patients who underwent either a one-day or a two-day stress-first protocol showed no differences in stress MBF. Significantly higher rest MBF values were calculated when a one day protocol was used (1.2 ± 0.5 ml/min/g vs. 1.0 ± 0.46 ml/min/g; p = 0.009, Fig. 3). Consequently MFR was lower in patients that underwent one day protocols (MFR NC 1.9 ± 1.0 ml/min/g vs. 2.2 ± 1.3 ml/min/g; p = 0.035; Fig. 3). This effect was not observed, when AC was used. A separate analysis for Tc-99 m-sestamibi and Tc-99 m-tetrofosmin showed that the global observation was driven by Tc-99 m-tetrofosmin scans (one day protocoll 1.4 ± 0.7 ml/min/g vs. two day protocol 1.0 ± 0.5 ml/min/g; p = 0.014) while in Tc-99 m-sestamibi scans no differences based on protocol use were detected (1.1 ± 0.4 ml/min/g vs. 1.0 ± 0.47 ml/min/g; p = 0.338).

### Use of attenuation correction lowers calculated MBF

**Fig. 4:**
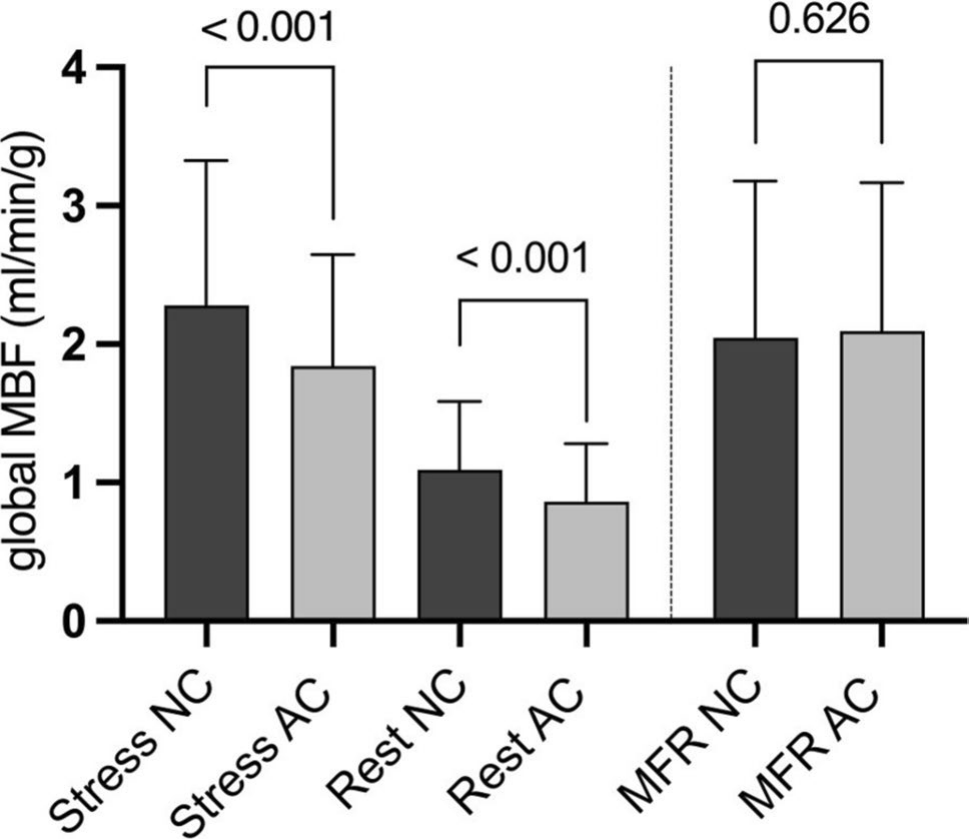
Use of attenuation correction (AC) systematically and significantly lowered the measured global myocardial blood flow (MBF) at stress and rest. No effect on myocardial flow reserve (MFR) was detected

**Fig. 5:**
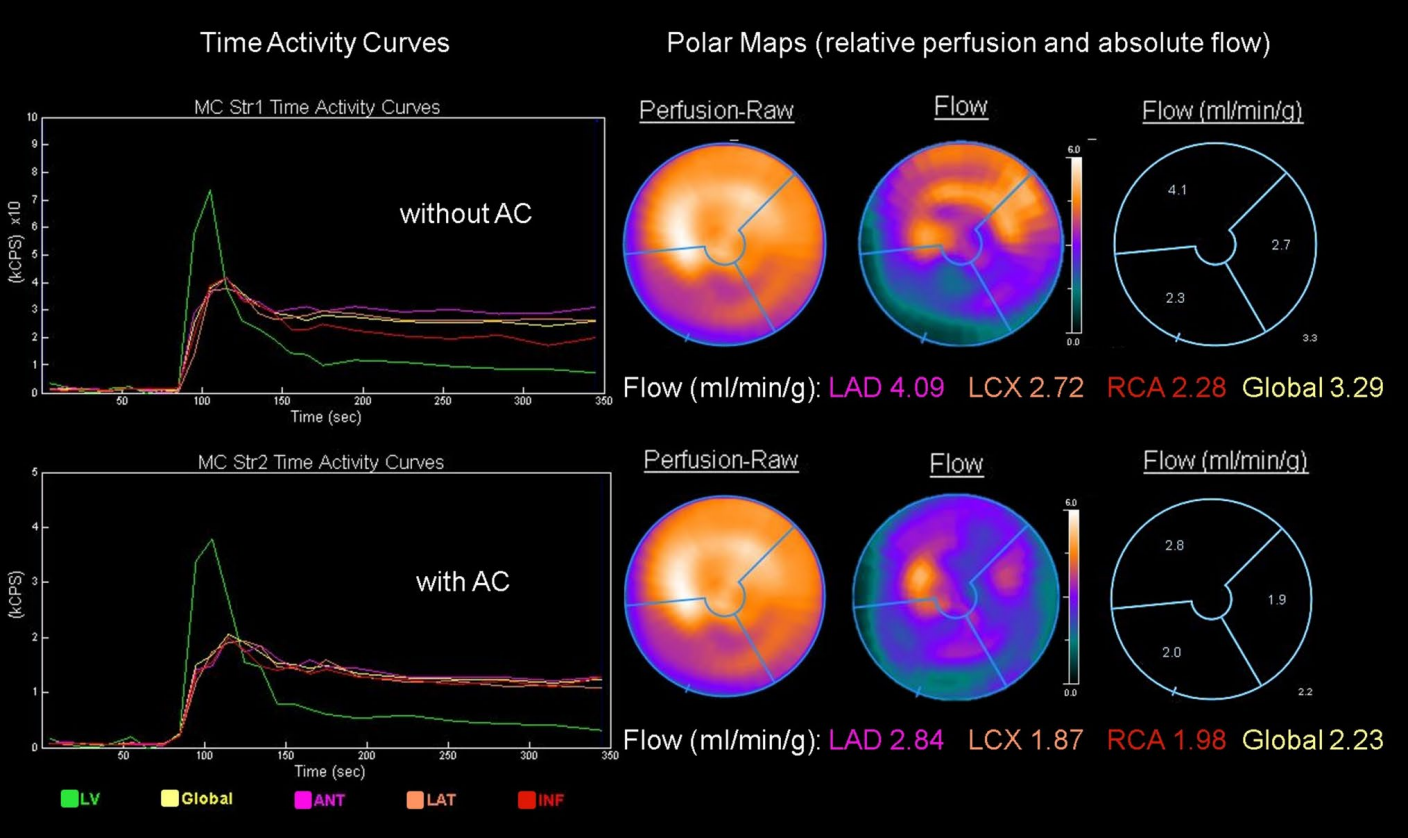
Patient example of a dynamic stress study analyzed in Corridor 4DM reserve software. Given are from left to right: Time-activity curves without and with attenuation correction (AC), perfusion polar maps, flow polar maps and calculated regional absolute quantitative flow measures (ml/min/g)

Mean global stress and rest MBF were significantly lower when AC was used (NC 2.3 ± 1.1 ml/min/g vs. AC 1.8 ± 0.8 ml/min/g; p < 0.001; NC 1.1 ± 0.5 ml/min/g vs. AC 0.9 ± 0.4 ml/min/g; p < 0.001, Fig. 4). Similar results were found for the regional analysis. However, there were no significant differences in the calculated MFR (NC 2.1 ± 1.1 ml/min/g vs. AC 2.1 ± 1.1 ml/min/g; p = 0.626). This finding aligns with the expectation that AC affects both stress and rest values proportionately, thereby preserving the calculated MFR. A patient example for calculation of global and regional stress MBF and effect of AC s given in Fig. 5.

### Presence of perfusion defects in standard static scans is associated with lower global and regional MBF

**Fig. 6:**
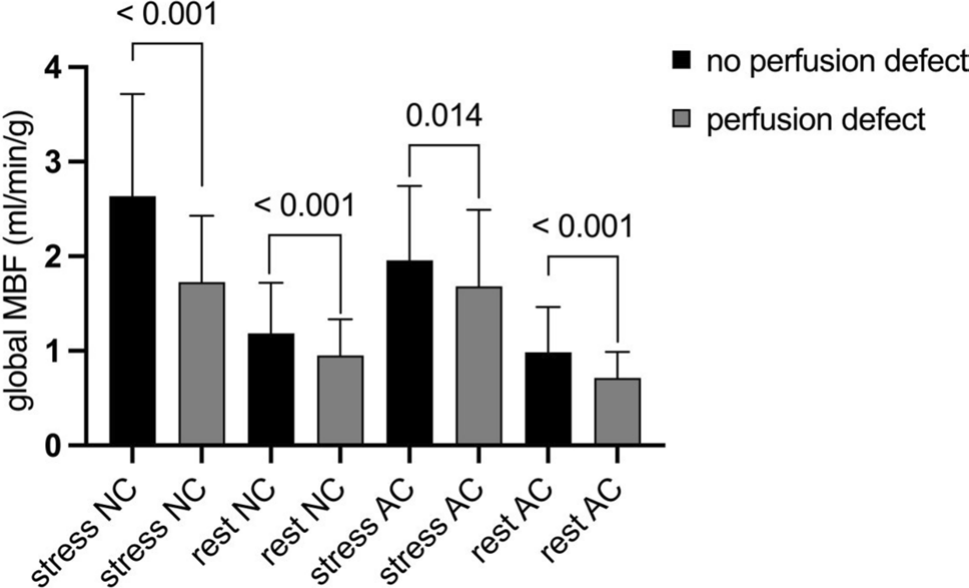
Presence of perfusion defects in standard static scans systematically lead to significantly lower global myocardial blood flow (MBF) at stress and rest in both non-attenuation (NC) and attenuation corrected (AC) flow measures

Relevant perfusion defects were defined as three or more segments with reduced perfusion in one coronary territory based on the 17-segments-AHA model, results of standard static scans are presented in Table 1. Presence of perfusion defects in static scans were associated with lower global stress and rest MBF (no defect: 2.6 ± 1.1 ml/min/g vs. defect 1.7 ± 0.7 ml/min/g; p < 0.001 and no defect 1.2 ± 0.5 ml/min/g vs. defect: 1.0 ± 0.4 ml/min/g; p < 0.001; Fig. 6). However, there were no significant differences for MFR between patients with and without defects in static scans (no defect p = 0.143; defect p = 0.234). Analogue results were found for AC MBF and MFR measures and for regional analysis.

### Summed defect scores derived from standard static scans correlate with global MBF

**Fig. 7:**
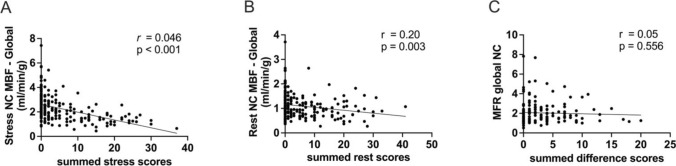
Significant inverse correlations were detected between (A) global stress myocardial blood flow (MBF) and summed stress scores (SSS) and (B) global rest MBF and summed stress scores (SRS). (C) No significant correlation was detected between global myocardial flow reserve (MFR) and summed difference scores (SDS)

Significant correlations between summed stress scores (SSS) and global stress MBF as well as summed rest scores (SRS) and global rest MBF were found (Fig. 7). However, no significant correlation was detected between summed difference scores (SDS = SSS-SRS) and MFR NC (r = 0.05, p = 0.556). Analogue correlations were calculated when AC was used.

### Lower LVEF is associated with lower MBF

**Fig. 8:**
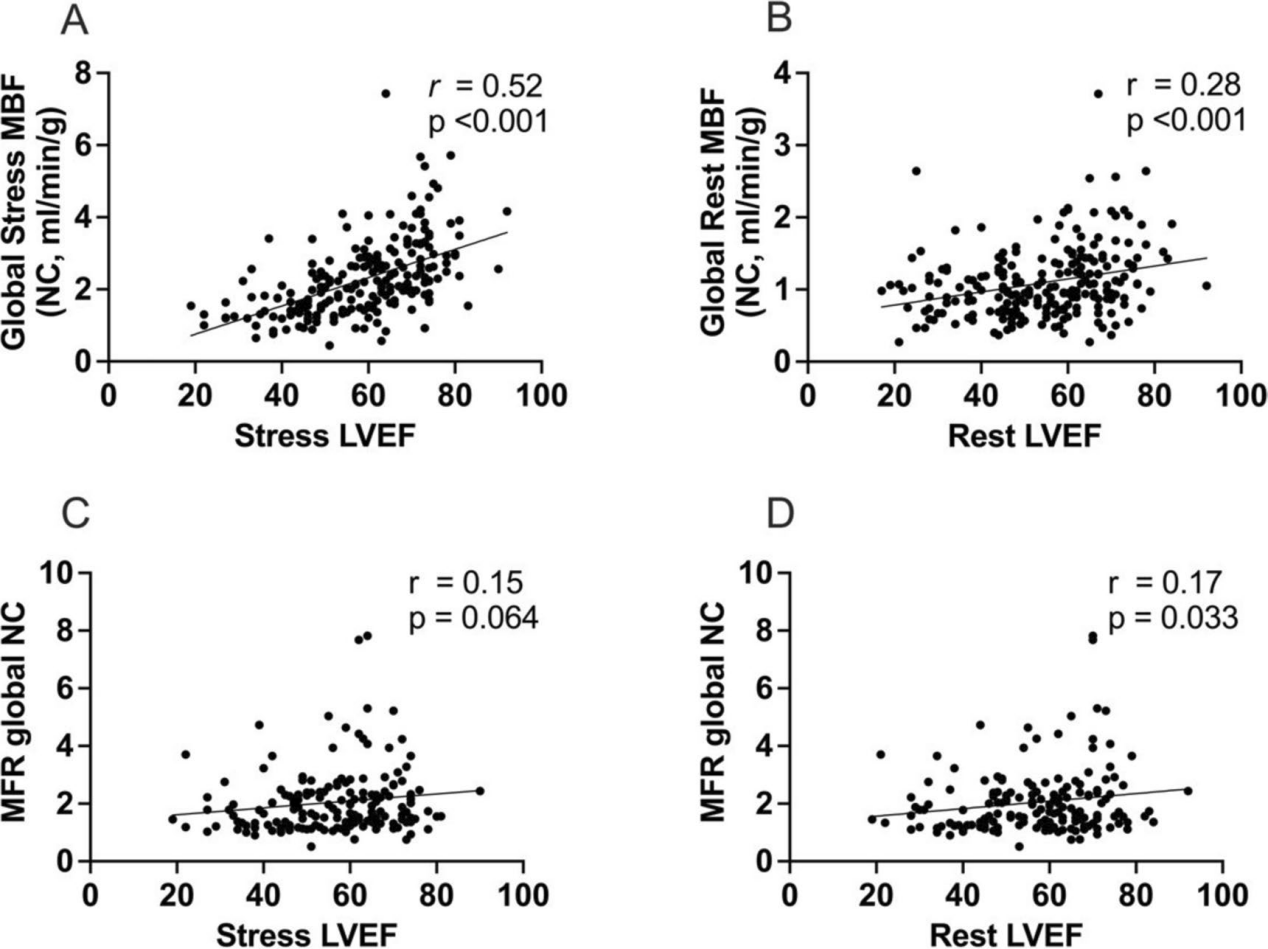
Significant correlations between (A) stress myocardial blood flow (MBF) and stress left ventricular ejection fraction (LVEF) and (B) rest MBF and rest LVEF were detected. Myocardial flow reserve (MFR) tended to correlate stress LVEF (C) and significantly correlated with rest LVEF (D)

Mean stress left ventricular ejection fraction (LVEF) was 58.8% and rest LVEF was 54.2%. Higher stress (r = 0.52, p < 0.001) and rest LVEF (r = 0.28, p < 0.001) correlated significantly with higher MBF measures (Fig. 8).

No significant correlation was detected between stress or rest LVEF and MFR.

### Influence of cardiovascular comorbidities on MBF

**Fig. 9:**
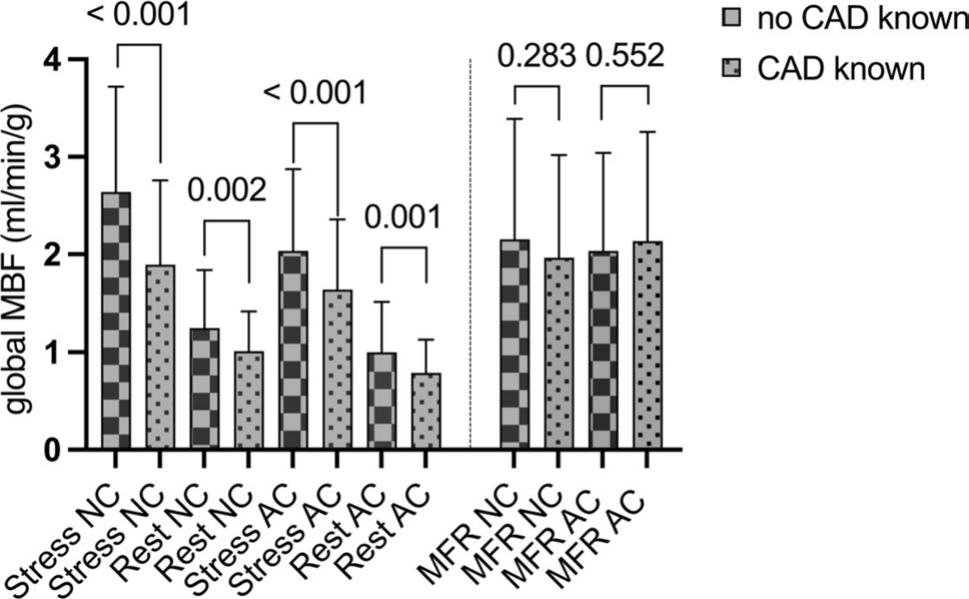
Patients with known coronary artery disease (CAD) had significantly lower global myocardial blood flow (MBF) at stress and rest in both non-attenuation (NC) and attenuation corrected (AC) flow measures. No significant reduction of myocardial flow reserve (MFR) was found

**Fig. 10:**
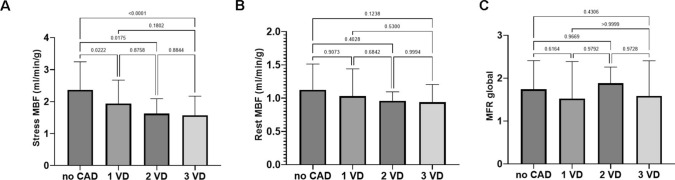
ANOVA comparisons of (**A**) stress myocardial blood flow (MBF), (**B**) rest MBF and (**C**) myocardial flow reserve (MFR) in patients without coronary artery disease (CAD), one-vessel disease (1 VD), two-vessel disease (2 VD) and three-vessel disease (3 VD). Significant differences between groups were only found under stress conditions, no relevant reduction of rest MBF or MFR was observed

Patients with known coronary artery disease (CAD) had a significantly lower global stress (1.9 ± 0.9 ml/min/g vs. 2.6 ± 1.1 ml/min/g, p < 0.001) and rest MBF (1.0 ± 0.4 ml/min/g vs. 1.3 ± 0.6 ml/min/g, p = 0.002) than patients without known CAD (Fig. 9). No significant reduction of MFR was seen in patients with history of CAD (p = 0.283). Stress MBF gradually declined the more vessels were known to be affected from CAD (Fig. 10), while rest MBF and MFR were not impacted.

Presence of cardiovascular risk factors (i.e. CAD, MI, hypertension, diabetes, obesity or smoking) lead to a significant reduction of calculated MBF. Patients with two or more known cardiovascular risk factors had significantly lower MBF than patients with less or without cardiovascular risk factors (global stress MBF: 2.1 ± 1.0 ml/min/g vs. 2.7 ± 1.0 ml/min/g, p < 0.001; global rest MBF: 1.0 ± 0.4 ml/min/g vs. 1.4 ± 0.6 ml/min/g, p = 0.004). For MFR, no significant difference was found (2.1 ± 1.2 ml/min/g vs. 1.8 ± 0.7 ml/min/g, p = 0.124).

## Discussion

Dynamic list mode imaging with CZT cameras now facilitates SPECT derived measurement of MBF and MFR [18]. In PET, being the gold standard, quantitative MBF assessment provides enhanced diagnostic precision, facilitating a comprehensive evaluation of myocardial perfusion abnormalities and aiding in risk stratification [24, 25]. The ability to measure flow reserve enables identification of subtle perfusion deficits and microcirculation abnormalities thus providing valuable prognostic information [22]. However, PET imaging is currently only feasible in larger hospitals or cardiovascular centers due to high costs and the need for an on-site cyclotron [26].

SPECT is a widely accessible technique, therefore calculating MBF and MFR from SPECT would be desirable. In this analysis, SPECT MBF quantification was performed as part of our clinical routine without any specific preselection. The detected mean stress and rest MBF and MFR were in range of expectation. Of note, few patients presented with extraordinary high flow measures which we re-analyzed but high measures without detectable reason persisted. We chose not to exclude these scans from our global analysis in order to show the high MBF variance. Expectedly, regionally highest variance was found in the RCA territory, which is the most complex region for SPECT most likely due to attenuation-related effects and infradiaphragmatic spillover. We showed that patients with high defect scores derived from standard static scans and patients with lower LVEF had impaired MBF. When employing Tc-99 m-sestamibi, there was slight yet significant increase in global stress in comparison to Tc-99 m-tetrofosmin analyzing all patients, however no differences were detected in the provided sub analysis. Slight differences between the tracers might be caused by the respective tracer extraction fraction [27]. No other relevant differences between the two used tracers were found.

Available data on patient history, laboratory values and cardiac interventions reflect a real-world situation where parts of information may be incomplete. Also, we did not systematically compare global or regional MBF with the current coronary status. In this regard, we still detected significant correlations between presence of cardiovascular comorbidities including CAD and calculated MBF and MFR. A known history of multi-vessel disease corresponded to worse stress MBF. Concordant to other studies specifically evaluating angiographic findings and SPECT MBF [16, 19, 28–31], our results further support credibility of this technique.

However, clinical utility of absolute quantitative MBF and MFR measures is not yet clear and several open questions have to be addressed to facilitate routine clinical use. It is well known that challenges accompany the acquisition of absolute quantitative MBF [32, 33]. The complexity of data processing, including the need for corrections in attenuation, scatter, and partial volume effects, pose hurdles in standardizing protocols across different centers. Dynamic image acquisition is more sophisticated than standard static acquisition. Patients have to be positioned without an injected full tracer dose and patient movement needs to be as small as possible. Limitations in hardware, specifically the spatial and temporal resolution of traditional SPECT systems, affect the accuracy of absolute MBF quantification, particularly in dynamic assessments [33].

For CZT-SPECT systems, CT AC is usually acquired on a separate camera since these systems are not equipped with CT. MBF calculation in PET however is always done with AC. The effect of CT AC on calculated SPECT MBF and MFR has been evaluated in few studies. We here observed a generally lower stress and rest MBF when AC was used but no effect on MFR was detected. This finding is consistent with results from Bailly et al. [34]. Other studies evaluated AC MBF results in comparison to PET and found partially inconclusive effects on global and regional MBF [17, 35]. Zavadovsky et al. found an improved correlation between stenosis severity and regional stress MBF and higher diagnostic accuracy for multivessel CAD when AC was used [23]. In summary, effects of AC have not yet been fully understood and therefore careful interpretation is obligatory.

In our clinical routine, both one-day and two-day protocols were used as appropriate. Ideally, for one-day protocols a stress / rest dosing ratio 1:2.5 or better is recommended [36]. The here observed average factor of 2.2 reflects real-world practice and strict dosing limitations in Germany. However, the average time interval between stress and resting studies for the one-day protocol was 2 h and 27 min, which should enable reliable measurements even at a slightly suboptimal stress / rest dosing ratio. We also corrected the second scan for residual activity. Still, we observed significantly higher rest MBF values and a consequently lower MFR. Interestingly, this effect was less pronounced, when AC or Tc-99 m-Sestamibi was used. This finding warrants further investigation. Ultimately, two-day protocols may be more suitable for accurate SPECT MBF and MFR calculation.

In summary, obtaining SPECT MBF and MFR is feasible in a clinical routine setting yielding values in range of expectation. The findings highlight the potential of CZT-SPECT for MBF and MFR quantification in routine practice, but also emphasize its limitations, including wide reference ranges and challenges in defining clinically meaningful thresholds such as an MFR cut-off of 2. Currently available analysis methods are time-consuming and technically demanding. There is a need for improved automated motion correction in order to bring the application to clinical routine use. Moreover, use of AC lowers calculated MBF and selection of protocol distinctly influences MBF and MFR results. Harmonization of imaging protocols between cardiovascular centers will improve inter-site comparability in the future [37]. Until then, absolute quantitative SPECT acquisition of MBF remains a possibility to enhance diagnostic value for specific clinical scenarios.

## Limitations

This is an observational study. Presented data is based on routinely acquired SPECT imaging without any study-related pre-selection of patients. Available data on patient history, laboratory values and cardiac interventions reflect a real-world situation where parts of information may be incomplete. To date there is no reliable reference standard for flow measurements derived from SPECT. Tc-99 m-labeled perfusion tracers have a high-count statistic and low extraction fraction at high flow rates which determines reduced contrast between stress and rest flow in comparison to values known from PET. No systematical comparison to PET data or findings from coronary angiography was included.

## Data Availability

No datasets were generated or analysed during the current study.
